# Long intergenic noncoding RNA smad7 (Linc‐smad7) promotes the epithelial‐mesenchymal transition of HCC by targeting the miR‑125b/SIRT6 axis

**DOI:** 10.1002/cam4.3515

**Published:** 2020-10-10

**Authors:** Lili Han, Lijun Jia, Ying Zan

**Affiliations:** ^1^ Department of Oncology The Second Affiliated Hospital College of Medicine Xi'an Jiaotong University Xi'an Shaanxi China

**Keywords:** Linc‐smad7, HCC, miR‐125b, SIRT6

## Abstract

Long intergenic noncoding RNA smad7 (Linc‐smad7) has been recently identified as a new long non‐coding RNA (lncRNA). However, the role of Linc‐smad7 in the tumourigenesis of human cancers remains unknown. This study uncovered that Linc‐smad7 was increased in HCC samples and HCC cell lines using RT‐qPCR assays. Furthermore, the overexpression of Linc‐smad7 indicated poor clinicopathological features and outcomes for HCC patients. In addition, Linc‐smad7 promoted HCC cells proliferation, migration, invasion and EMT, as determined by MTT, colony formation, Transwell assays and western blot analysis. Functionally, it was demonstrated that Linc‐smad7 could bind with microRNA‑125b (miR‑125b), and the restoration of miR‑125b rescued the promoting effects of Linc‐smad7 on HCC cells. Finally, it was observed that sirtuin 6 (SIRT6) was positively regulated by Linc‐smad7 in HCC as the direct target of miR‑125b, and decreased SIRT6 reversed the effects of Linc‐smad7 on promoting HCC. In conclusion, the current study first identified Linc‐smad7 is increased in HCC, facilitating HCC cells proliferation, migration, invasion and EMT via regulating the miR‑125b/SIRT6 axis.

## Graphical abstract

The current study uncovered that Linc‐smad7 was upregulated in HCC tissues and HCC cell lines. Furthermore, overexpression of Linc‐smad7 indicated poor clinical features and outcomes in HCC patients. In addition, overexpression of Linc‐smad7 promoted HCC cell proliferation, invasion, migration and EMTby modulating the miR‐125b/SIRT6 axis.

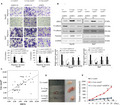

## 1 INTRODUCTION

Hepatocellular carcinoma (HCC) is one of the major cause of cancer‐related mortality in the worldwide.^1^ In spite of continuing efforts have been made in terms of treatment over the past few decades, the current treatment methods, including surgical resection, molecular targeted therapy and immunotherapy, are still only effective for a subset of patients.^2^ The long‐term survival in the overall population of HCC patients remains disappointing.^3^ Metastasis and recurrence after surgical resection are frequently observed in clinical settings and are one of the primary causes for the high mortality rate of HCC. Thus, a more intimate understanding of the underlying molecular mechanisms could be pivotal to uncover novel molecules to counteract the metastasis of HCC.

Epithelial‐mesenchymal transition (EMT), making tumour cells lose their original characteristics of adhesion and transform into cells with mesenchymal characteristics, is closely associated with HCC metastasis.^4^, ^5^, ^6^ The extremely complex network involved in the regulation of EMT has not been fully elucidated.^7^, ^8^ Long non‐coding RNAs (lncRNAs), a group of RNAs consisting of >200 bp without protein‐coding capacity, act as pivotal regulators in multiple cellular processes closely correlate with the occurrence of human cancers by regulating their target gene expression.^9^, ^10^, ^11^ Accumulating evidence suggest that lncRNAs, such as Linc‐DYNC2H1‐4,^12^ Linc‐RoR,^13^ Linc‐ITGB1,^14^ Linc‐ITGB1^15^ and Linc‐DYNC2H1‐4,^16^ regulate metastasis and EMT in human cancers. These studies suggest that regulation of lncRNAs may provide a novel therapeutic choice for human cancer treatment. Long intergenic noncoding RNA smad7 (Linc‐smad7) is a recently identified promoter of skeletal muscle development. However, the role of Linc‐smad7 in HCC remains unknown. Thus, the objective of this current study was to study the function of Linc‐smad7 in HCC.

In this study, we evaluated the expression pattern and function of Linc‐smad7 in HCC. The data suggested that Linc‐smad7 is increased in HCC and positively regulate SIRT6 expression by inhibiting microRNA‐125b in HCC cells. Collectively, these results demonstrated the novel oncogenic role of Linc‐smad7 in HCC.

## 2 MATERIALS AND METHODS

### 2.1 Patients and tissue samples

This study is approved by The Ethics Committee of the Medical School of Xi'an Jiaotong University. HCC tissue and paired noncancerous tissue samples (>2 cm away from the edge of tumour) were gathered from HCC patients received a surgical resection between May 2010 and May 2011 at The Second Affiliated Hospital of Xi'an Jiaotong University. HCC patients were diagnosed histologically and had not received immunotherapy and chemotherapy before operations. Table 1 showed the clinicopathological feature of these HCC. The samples were immediately frozen and stored in liquid nitrogen after surgery.

**Table 1:** Association between Linc‐samd7 expressions and clinicopathological features in HCC

Variable	Total no. of patients *n* = 50	Linc‐smad7		*p*
		Low expression	High expression	
Age (years)				0.100
<50	20 (40%)	8	12	
≥50	30 (60%)	5	25	
Gender				0.248
Female	11(22%)	1	10	
Male	39(78%)	12	27	
HBsAg				0.542
Positive	32 (64%)	8	24	
Negative	18(36%)	5	13	
AFP (ng ⁄ mL)				0.214
<400	9 (18%)	4	5	
≥400	41(82%)	9	32	
Tumour size (cm)				**0.014** **
<5	16(32%)	8	8	
≥5	34(68%)	5	29	
Tumour multiplicity				0.328
Single	32(64%)	10	22	
Multiple	18(36%)	3	15	
Differentiation				0.554
Well–moderate	22(44%)	6	16	
Poor–undifferentiation	28(56%)	7	21	
Microscopic vascular invasion				**0.000** **
Yes	20(40%)	12	8	
No	30(60%)	1	29	
Stage				**0.038** *
I‐II	33(66%)	12	21	
III‐IV	17(34%)	1	16	

* *p* < 0.05

** *p* < 0.01

### 2.2 Immunohistochemistry (IHC)

The immunohistochemistry assay was carried out according to the steps reported previously.^17^ The primary antibodies, including anti‐E‐cadherin antibody (bs‐1016R), anti‐Vimentin antibody (bs‐1172R) and anti‐N‐cadherin antibody (bs‐8533R) were bought from Beijing Biosynthesis Biotechnology. The secondary antibody (cat. no. A0279; 1:200) was purchased from Beyotime Institute of Biotechnology. Finally, the images were captured using Leica TCS SP2 confocal laser‐scanning microscope.

### 2.3 The RT‐qPCR assay

Total RNA was prepared from tissue samples or cells using RNeasy reagent (Qiagen, Shanghai, China). A reverse transcription kit (Takara, Otsu, Shiga, Japan) was then used to reverse transcribe RNA samples into cDNA. SYBR Premix Ex Taq (Takara) was used to perform RT‐qPCR. U6 and GAPDH RNA expression were used as the endogenous control for miR‐125b or lncRNA, respectively. The relative expression levels of Linc‐smad7 and miR‐125b were calculated against the expression of endogenous control using the relative quantification method (2–^ΔΔCt^ approach). Table 2 showed the primers sequences. Each measurement was analysed in triplicate.

**Table 2:** Information of the qPCR primer sequences and silencing RNA sequences

RT‐qPCR primer name	Sequence (5’−3’)
Linc‐smad7	(Forward) TTGGCCAATCAGGAACAT (Reverse) AATGCATGCTGAGTGTCA
miR−125b	(Forward) ACCCAGTGCGATTTGTCA (Reverse) ACTGTACTGGAAGATGGACC
SIRT6	(Forward)AGTCTTCCAGTGTGGTGTTCC (Reverse) TCCATGGTCCAGACTCCGT
E‐cadherin	(Forward) TGAAGGTGACAGAGCCTCTGG (Reverse) TGGGTGAATTCGGGCTTGTT
Vimentin	(Forward) GACGCCATCAACACCGAGTT (Reverse) CTTTGTCGTTGGTTAGCTGGT
N‐cadherin	(Forward) TCAGGCGTCTGTAGAGGCTT (Reverse) ATGCACATCCTTCGATAAGACTG
U6	(Forward) CTCGCTTCGGCAGCACA (Reverse) AACGCTTCACGAATTTGCGT
GAPDH	(Forward) GCACCGTCAAGGCTGAGAAC (Reverse) ATGGTGGTGAAGACGCCAGT

### 2.4 Cell culture and transfection

Huh7, MHCC‐97L, Hep3B, MHCC‐97H and THLE‐2 cells used in this study were bought from FuHeng Cell Center (Shanghai, China). The Dulbecco's modified Eagle medium DMEM (Sigma Aldrich, Germany) added with 10% foetal bovine serum (FBS, Gibco, Thermo Fisher Scientific, Inc.) was used to culture these cells. miR‐125b mimics (5'‑ UCCCUGAGACCCUAACUUGUGA ‑3’), negative control mimic (miR‐125b NC, 5'‑UAGCUUAUCAGACUGAUGUUGA‑3’); miR‐125b inhibitor (5'‑CAUGGCUGCGUCCCGUGA ‑3’) and the control (5'‑CUAGAGCCACGAUACGG‑3’) were bought from Gene Copoeia (Guangzhou, China). The lentivirus vector overexpressing Linc‐smad7 (Lv‑Linc‐smad7) and its negative control vector (Lv‐NC), shRNA‐encoding lentiviruses targeting Linc‐smad7 (sh‐Linc‐smad7) and its negative control vector (NC), as well as Linc‐smad7‐WT and Linc‐smad7‐MUT vectors used in the study, were constructed by GenePharma (Shanghai, China). The indicated vectors were treated into HCC cells when HCC cells reached 40%‐50% density following the manufacturer's protocol. A total of 25 µg vectors in 2 ml of medium with 1 ml of enhanced transfection solution and 5 µg of polybrene (GenePharma, Shanghai, China) were added into HCC cells. Subsequently, puromycin with the concentration of 2 µg/ml was added to each well to select the stable Linc‐smad7 overexpressing or downregulating cells. For transfecting miR‑125b mimics or inhibitors, a total of 200 nM mimics or inhibitors or were added to 2 ml serum‐free DMEM dissolved 20 µl Lipofectamine 2000 and then added to each well. The specific short hairpin sh‐SIRT6 vector and its control vectors were bought from Genepharma. The vectors were treated into HCC cells using Lipofectamine 2000. G418 (Gibco; Thermo Fisher Scientific, Inc.) was used to establish stable clones. These transfection efficiency were assessed by RT‐qPCR or western blotting assays.

### 2.5 Western blot analysis

RIPA buffer (Thermo Fisher Scientific, Inc.) was used to lyse the tissue samples or cell lines were. BCA bought from Cell Signaling Technology, Inc. (Danvers, MA, USA) were used to measure the concentrations of protein. The protein sample (20 μg) separated by 7.5 ~ 12.5% SDS polyacrylamide gels (Bio‐Rad Laboratories, Inc., Hercules, CA) was transferred onto PVDF membranes (Millipore, Darmstadt, Germany), which were then incubated with indicated primary antibodies at 4°C for 12 h after blocked in 5% milk. After washing with PBS‐T buffer, membranes were treated with secondary antibodies at 37°C for 1.5 ~ 2 hours. A bio‐imaging system (DNR Bio‑Imaging Systems) was used to detect the signals. The primary antibodies anti‐E‐cadherin (1:1000, #3195), anti‐Vimentin (1:500, #5741), anti‐N‐cadherin (1:500, #13116) antibodies were bought from Cell Signaling Technology corporation. GAPDH (1:5000, G5262) was purchased from Sigma. The goat anti‐rabbit secondary antibody (1:10000, bs‑40295G‑IRDye8) and the goat anti‐mouse secondary antibody (1:5000, bs‐40296G‐IRDye8) were purchased from Beijing Bioss Biotechnology.

### 2.6 MTT assays

HCC cells were added to a 96‐well plate (500‐600 cells per well) and incubated in 100 µl DMEM added with 10% FBS. At the indicated times (24, 48 and 72 h), HCC cells in each well were treated with 10 μl MTT solution (5 mg/ml) and then incubated at room temperature. After 4 h, the fresh medium containing 200 µl of dimethyl sulfoxide (DMSO, 1 mg/ml) was added in instead the medium. Finally, a Bio‑Rad Laboratories plate reader (Hercules, USA) was used to assess cell viability by detecting absorbance at a wavelength of 492 nm.

### 2.7 Cell migration and invasion assays

The upper chamber was treated with HCC cells (3 × 10^4^) in 200 μl medium without FBS. While, the bottom chamber was treated with 600 µl media with 10% FBS. After 36‐48 h of incubation, the cells remaining inside the upper chamber were removed, while the cells that had migrated through the membrane were washed and fixed by methanol, and then stained with crystal violet solution (0.1% Amresco, Solon, OH, USA). Finally, a microscope (Olympus Corporation, Tokyo, Japan) was used to count the number of migrated cells.

Invasion assay has the same steps of migration assay, except the membrane of the inserts was coated with 20 µg Matrigel (200 mg/ml, Becton Dickinson Biosciences, USA).

### 2.8 Colony forming assays

HCC cells (200 per well) at the exponential growth phase were added into a 6‐well plate with 2 ml complete medium. After incubated for 10 days, the colonies were fixed with methanol and stained by 0.1% crystal violet solution at 37°C. The number of colony forming units (those containing ≥50 cells) was assessed by an inverted microscope (Nikon, Tokyo, Japan, x40). All experiments were carried out in triplicate.

### 2.9 Luciferase reporter assays

HCC cells (8x10^4^ per well) in a 24‐well plate were co‐transfected with miR‑125b mimics, Renilla luciferase reporter plasmid, or Linc‐smad7‐WT or Linc‐smad7‐MUT luciferase reporter plasmid (GenePharma Co., Ltd., China) using Lipofectamine 2000 following the manufacturer's protocol. After transfection for 48 h, the Dual‐Luciferase Reporter Assay System kit (Promega, Madison, USA) was used to measure the luciferase activities. The Renilla luciferase activity served as an internal control was used to assess the relative firefly luciferase activity.

### 2.10 RNA pull‐down assay

First, full‐length Lin‐smad7‐WT and Linc‐smad7‑MUT sequences were amplified using PCR kits (Takara Bio, Inc.). Subsequently, the restriction enzyme BamHI was used to linearize the plasmids. The biotin‐labelled RNAs were produced with a biotin RNA labelling kit and T7 RNA polymerase (cat. no. C11002‑1; Guangzhou RiboBio Co., Ltd.). Then, the biotin‐labelled Linc‐smad7‐WT or Linc‐smad7‑MUT was incubated with probe‐coated beads (cat. no. 65002; Invitrogen; Thermo Fisher Scientific, Inc.) at 4°C for 12 h. Finally, the pulled‐down RNA was measured by RT‐qPCR analysis. The sequence of Linc‐smad7‐WT is CGGGACTCGGCGGCGTCTTG; the sequence of Linc‐smad7‐MUT is CGATCGTCGGCGGCGTCTTG.

### 2.11 Animal model assays

The animal model assays in this study were approved by the Experimental Animal Ethical Committee of the Medical School of Xi'an Jiaotong University. The BALB/c male mice (four groups, six in each group) were bought from SLAC Laboratory Animal Company (SCXK2007‐004). HCC cells transfected with Lv‐Linc‐smad7/Lv‐control or Lv‐Linc‐smad7+shSIRT6 vectors (1 × 10^7^ suspended in 150 µL media without serum) were injected into the right flank of the mice. The tumourigenesis was observed daily and the tumour volume was calculated with the formula length × width^2^ × 0.5. After 1 month, the mice were sacrificed, and the tumours were surgically removed, the tissues were fixed in 4% paraformaldehyde.

### 2.12 Statistical analysis

SPSS statistical software (version 19.0; IBM Corp.) was used to perform statistical analyses. All experiments were repeated in triplicate, and the data are expressed as the mean ±standard deviation (SE). The differences between two groups were assessed with Student's t‐test. One‐way analysis of ANOVA with Dunnett multiple comparisons test and Tukey test were used in comparing the mean of multiple experimental groups and the mean of control group, or multiple comparisons between all groups, respectively. The relationship between Linc‐smad7 and HCC patient clinicopathological feature was analysed using Chi‐square tests (χ2). Spearman correlation was used to evaluate the correlation between Linc‐smad7 and miR‐125b in clinical samples from HCC patients. Kaplan‐Meier curves and log‐rank tests were used to measure the survival of different groups. *p＜0.05 from a two‐sided test was used to indicate a statistically significant difference.

## 3 RESULTS

### 3.1 Linc‐smad7 is upregulated in HCC tissues and HCC cells

**Figure 1:**
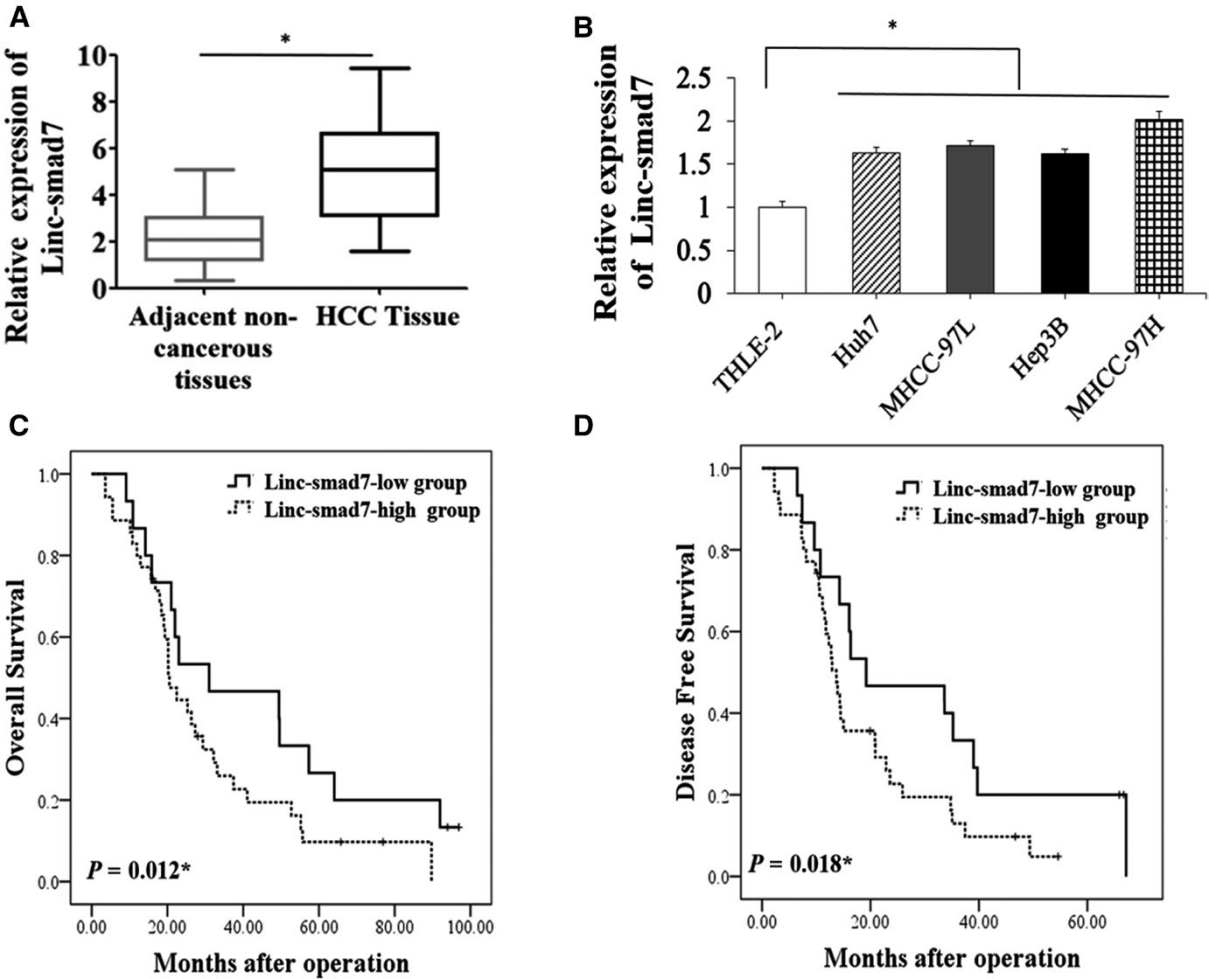
Upregulation of Linc‐smad7 in HCC tissues and HCC cells. (A) The expression of Linc‐smad7 in HCC samples was analysed by RT‐qPCR (*p < 0.05). (B) Linc‐smad7 expression levels in various human HCC cell lines and the human normal hepatocyte cell line THLE‐2(*p < 0.05). (C) Comparison of OS between HCC patients with high and low Linc‐smad7 expression levels (*n* = 50) (*p < 0.05). (D) Comparison of DFS between HCC patients with high and low Linc‐smad7 expression levels (n = 50) (*p < 0.05).

To explore the expression pattern of Linc‐smad7 in HCC, we first measured the expression of Linc‐smad7 in 50 pairs of HCC samples and adjacent non‐tumour tissues from HCC patients. The results of RT‐qPCR analysis indicated that Linc‐smad7 was much higher in the HCC tissues than that in the non‐tumour tissues (*p *< 0.01, Figure 1A). We further detected Linc‐smad7 expression in HCC cells and normal human liver cells (THLE‐2). The RT‐qPCR results in vitro are consistent with the results in the clinical samples, Linc‐smad7 expression was observably upregulated in HCC cell lines compared with THLE‐2 cells, (*p *< 0.05, Figure 1B).

We also analysed the clinical significance of the increased Linc‐smad7 expression in HCC patients. High Linc‐smad7 expression level was closely associated with larger HCC tumour size (*p *= 0.014), advanced TNM stage (*p* = 0.038) and microscopic vascular invasion (*p *< 0.001) of HCC. While we did not observed the association between Linc‐smad7 and HBV (Table 1). Subsequently, Kaplan‐Meier analysis was performed to evaluate the prognostic value of Linc‐smad7 expression levels for HCC patients. The results showed that compared with HCC patients in Linc‐smad7‐low group, those in Linc‐smad7‐high group had shorter overall survival (OS) (*p *< 0.05, Figure 1C) and disease‐free survival (DFS) (*p *< 0.05, Figure 1D).The results suggested that increased Linc‐smad7 expression may be closely associated with frequent recurrence of HCC after surgery.

### 3.2 Linc‐smad7 promotes HCC cells proliferation, migration and invasion.

**Figure 2:**
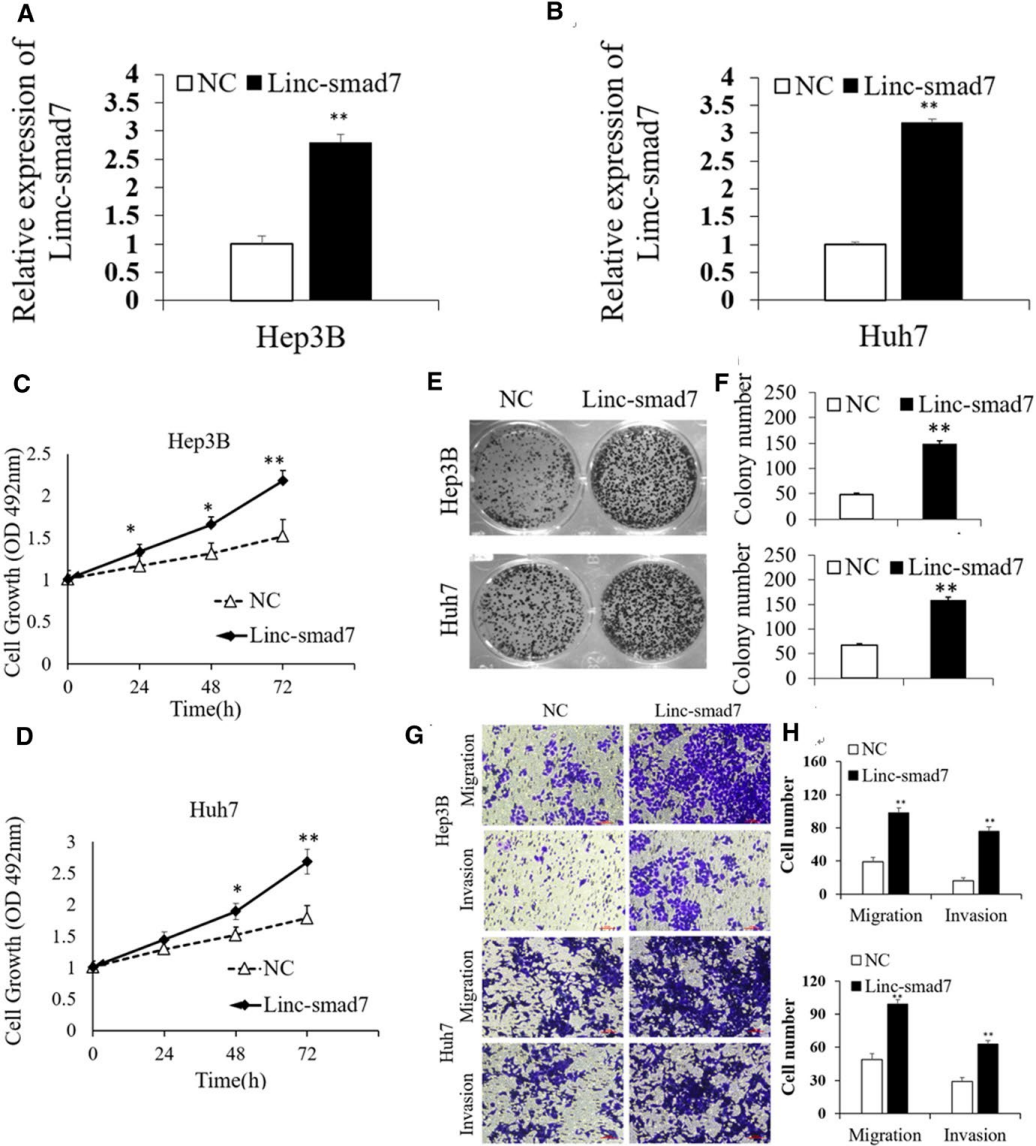
Linc‐smad7 promoted HCC cells proliferation, migration and invasion. (A,B) Linc‐smad7 expression was increased in Hep3B cells and Huh7 cells transfected with overexpressing Linc‐smad7 vector compared with that in cells transfected with the NC vector (**p < 0.01). (C,D) MTT assays showed that Linc‐smad7 promoted HCC cell proliferation (*p < 0.05). (E,F) Linc‐smad7 promoted colony formation of HCC cells (**p < 0.01). (G,H) Transwell assays indicated that Linc‐smad7 promoted HCC cells migration and invasion (**p < 0.01). The invaded cells were quantified by counting the cells in 10 random fields (magnification, 200×). Linc‐smad7 group: HCC cells transfected with Linc‐smad7 overexpression vector; **NC group:** HCC cells transfected with control vector.

We established a stable Linc‐smad7‐overexpressing Huh7 and Hep3B cell lines using lentivirus infection (Linc‐smad7 group) to help assess the function of Linc‐smad7 in HCC cells. RT‐qPCR assays showed that Linc‐smad7 expression was higher in HCC cells in Linc‐smad7 group than those in NC group (HCC cells transfected with the Lv‐control vector) (*p *< 0.01, Figure 2A and B). The results of MTT assays demonstrated that the proliferation of Huh7 and Hep3B cells was markedly enhanced in the Linc‐smad7‐overexpressing group (*p *< 0.05, Figure 2C and D). The colony formation showed that increased Linc‐smad7 significantly enhanced the colony formation ability of HCC cells (*p* < 0.01, Figure 2E and F). We next carried out Transwell assays to evaluate the role of Linc‐smad7 in HCC cell migration and invasion. The results suggested that overexpression of Linc‐smad7 significantly facilitated migration and invasion of HCC cell (*p *< 0.01, Figure 2G and H). These results further demonstrated that Linc‐smad7 serves as an oncogene in HCC.

### 3.3 Linc‐smad7 promotes HCC EMT.

**Figure 3:**
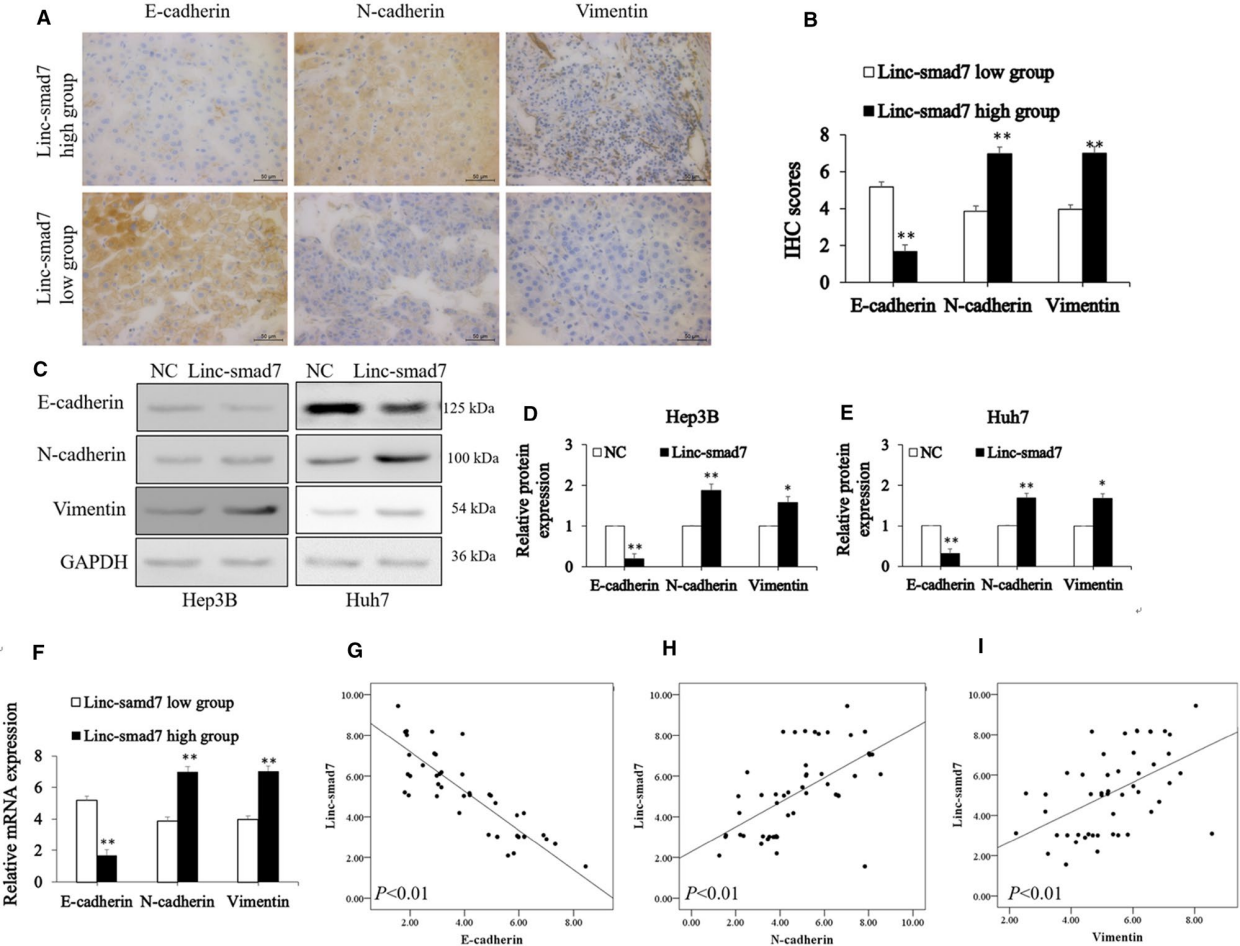
Linc‐smad7 promoted HCC EMT. (A,B) Immunohistochemistry of E‐cadherin, N‐cadherin and Vimentin in HCC tissues with high and low Linc‐smad7 expression (**p < 0.01; magnification, x400). (C,D,E) Western blot analysis of E‑cadherin, N‐cadherin and Vimentin expression following the overexpression of Linc‐smad7 in HCC cells (*p < 0.05, **p < 0.01). (F) RT‐qPCR analysis of E‑cadherin, N‐cadherin and Vimentin expression following the overexpression of Linc‐smad7 in HCC cells (**p < 0.01). (G,H,I) The correlation of Linc‐smad7 expression and E‑cadherin expression (Spearman's r = −0.870; *p* < 0.01), N‐cadherin expression (Spearman's r = 0.592; *p* < 0.01) or Vimentin expression (Spearman's r = 0.512; *p* < 0.01).

EMT is recognized as the initial step and an important mechanism of metastasis of human cancers. Thus, we speculate that Linc‐smad7 promotes HCC cells migration and invasion via influencing EMT. To confirm our speculation, we performed an IHC assay to assess the expression of Vimentin, N‐cadherin and E‐cadherin in HCC samples. The results showed that E‐cadherin expression was obviously lower in HCC tissues from Linc‐smad7‐high group than in those in Linc‐smad7‐low group. The expression of N‐cadherin and Vimentin showed the opposite result (*p* < 0.01, Figure 3A and B). Western blot assay showed increased Linc‐smad7 enhanced N‐cadherin and Vimentin expression, while significantly inhibited E‐cadherin expression (*p* < 0.05, Figure 3C‐E). We also transfected Linc‐smad7‐overexpressing (Linc‐smad7 group) or its negative control lentivirus vectors into THLE‐2 cells (Figure S1A), then found the downregulation of E‐cadherin and upregulation of N‐cadherin, Vimentin in RNA and protein expression levels (*p* < 0.05, FigureS1B and C). We further knocked down Linc‐smad7 upon the lentiviruses approaches (sh‐Linc‐smad7 group) in Hep3B and Huh7 cells (Figure S2A), and confirmed that decreased Linc‐smad7 inhibited N‐cadherin and Vimentin expression, while promoted E‐cadherin expression (*p* < 0.01, Figure S2B and C). In addition, we measured the mRNA expression of N‐cadherin, Vimentin and E‐cadherin in HCC samples using the RT‐qPCR assay (*p* < 0.01, Figure 3F). Spearman correlation analysis using RT‐qPCR showed that Linc‐smad7 expression was negatively correlated with E‐cadherin expression (*r* = −0.870, *p* < 0.01, Figure 3G), positively correlated with N‐cadherin (*r* = 0.592, *p* < 0.01, Figure 3H) and Vimentin expression (*r* = 0.512, *p* < 0.01, Figure 3I). Both the data from HCC samples and cells strongly indicated that Linc‐smad7 promoted EMT in HCC.

### 3.4 Linc‐smad7 is inversely correlated with miR‑125b in HCC

Given that Linc‐smad7 can regulate genes, including E‐cadherin, N‐cadherin and Vimentin, which are pivotal for EMT, we speculated that Linc‐smad7 may inhibit some miRNAs, consequently downregulating their target mRNA transcripts. The miR‐125 family has been proven to have a significant role in various human cancers as either repressors or promoters that regulate cell proliferation, apoptosis and cellular differentiation.^18^, ^19^ Several studies revealed that miR‐125b inhibited HCC metastasis by regulating its targets genes and downstream signals.^20^, ^21^, ^22^

**Figure 4:**
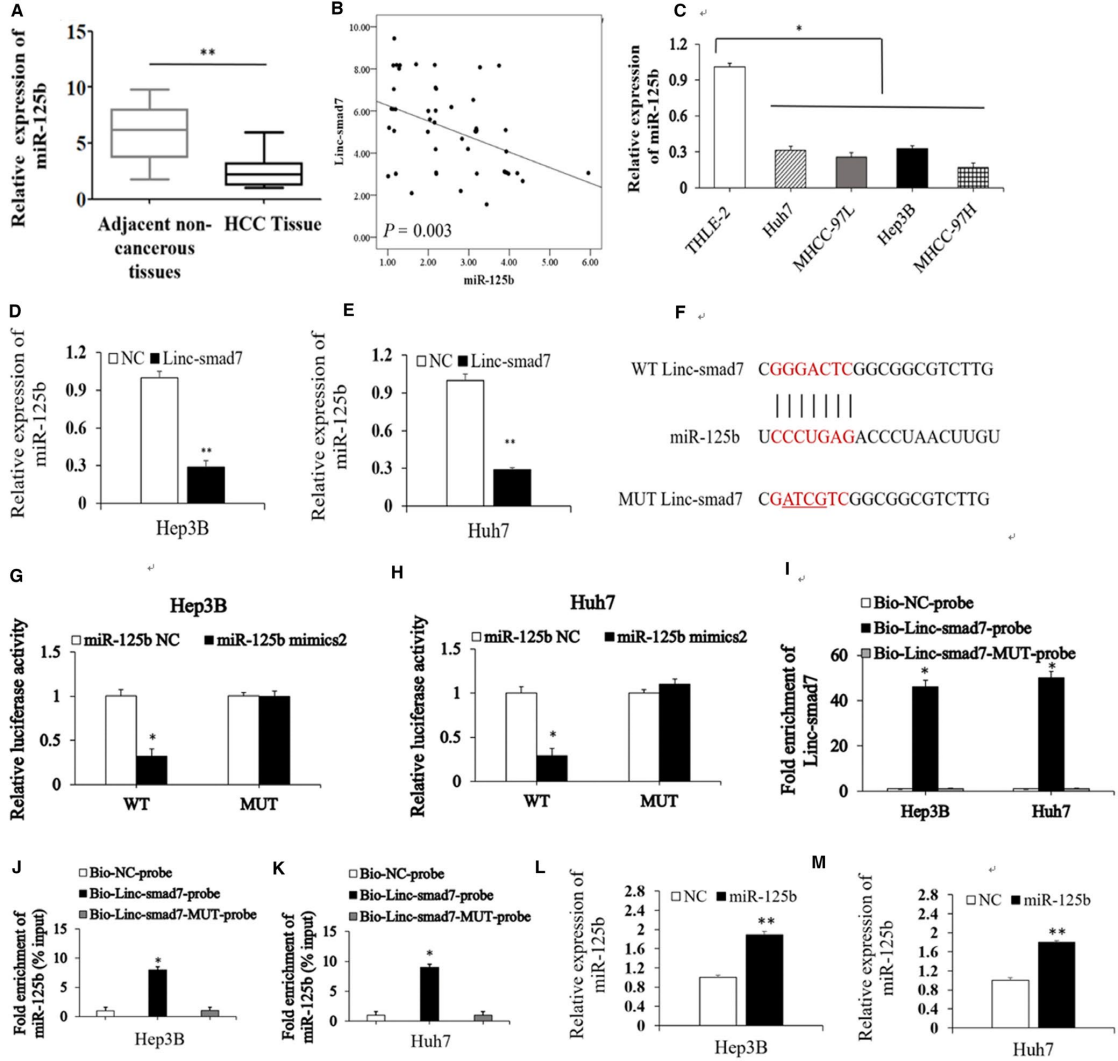
Linc‐smad7 directly binds with miR‑125b in HCC cells. (A) The expression of miR‐125b in HCC samples was analysed by RT‐qPCR (**p < 0.01). (B) The correlation of miR‐125b and Linc‐smad7 levels (*r* = −0.418; *p* = 0.003). (C) miR‐125b expression levels in various human HCC cell lines and THLE‐2 cells measured by RT‐qPCR (*p < 0.05). (D, E) The expression of miR‐125b in HCC cells transfected with Linc‐smad7 vector or NC vector (**p < 0.01). (F) The binding sequence in the linc‐smad7 transcripts was found to pair with miR‐125b. (G,H) Relative luciferase activity in Hep3B and Huh7 cells co‐transfected with Linc‐smad7‑WT or Linc‐smad7‑MUT luciferase plasmid and miR‑125b NC or mimics. (I) Detection of Linc‐smad7 in an RNA pull‐down assay with Bio‑Linc‐smad7, Bio‑Linc‐smad7‑MUT or Bio‑NC probes**.** (J,K) Detection of miR‑125b in an RNA pull‐down assay with Bio‑Linc‐smad7, Bio‑Linc‐smad7‑MUT or Bio‑NC probes in Hep3B and Huh7 cells. (L,M) miR‐125b expression was increased in Hep3B cells and Huh7 cells transfected with miR‐125b mimics compared with that in cells transfected with mimic‐NC control (**p < 0.01).

A recent study revealed that Linc‐smad7 promoted muscle regeneration and myoblast differentiation by sponging miR‐125b.^23^ Notably, the RT‐qPCR analysis in this study revealed that miR‑125b expression was downregulated in HCC tissues (*p* < 0.05; Figure 4A) and were negatively associated with Linc‐smad7 expression (*r* = −0.418, *p* = 0.003, Figure 4B **)**. Furthermore, miR‐125b expression was also decreased in HCC cells compared with THLE‐2 cells (*p* < 0.05, Figure 4C). To confirm our speculation, we performed RT‐qPCR to measure miR‐125b expression in Linc‐smad7 overexpressing HCC cells. The results of RT‐qPCR assay showed that the expression of miR‐125b was markedly lower in Linc‐smad7‐overexpressing Hep3B and Huh7 cells than that in control vector‐transfected cells (*p* < 0.05, Figure 4D and E). We also evaluated miR‐125b expression in Hep3B and Huh7 cells transfected with sh‐Linc‐smad7 vectors, and found that miR‐125b expression was increased followed with the knockdown of Linc‐smad7 (*p* < 0.01, Figure S3A). On the contrary, Linc‐smad7 expression was not changed in Hep3B and Huh7 cells treated with miR‐125b inhibitors (*p* < 0.01, Figure S3B). Also, miR‐125b was confirmed to bind sequences in the linc‐smad7 transcript (Figure 4F). These results demonstrated that Linc‐smad7 inhibited miR‑125b expression in HCC.

### 3.5 Linc‐smad7 directly binds with miR‑125b in HCC cells.

To confirm whether Linc‐smad7 could directly bind with miR‑125b, we constructed Linc‐smad7‐WT and Linc‐smad7‐MUT vectors. Dual‐luciferase reporter assay results demonstrated that the relative luciferase activities in HCC cells co‐transfected with Linc‐smad7‐WT and miR‐125b mimics were significantly reduced compared with those in HCC cells transfected with NC vector (*p* < 0.05, Figure 4G and H). While, the relative luciferase activities in HCC cells co‐transfected with miR‐125b mimics and Linc‐smad7‐MUT did not decrease significantly (*p* < 0.05, Figure 4G and H). In addition, biotin‐labelled RNA pull‐down assays showed that the bio‑Linc‐smad7 probe could pull down miR‑125b in HCC cells, but the bio‑Linc‐smad7‑MUT‑probe did not affect miR‑125b in HCC cells (*p* < 0.05, Figure 4I‐K**)**. Compared with that in cells transfected with mimic‐NC control, miR‐125b expression was increased in Hep3B cells (Figure 4L) and Huh7 cells (Figure 4M) transfected with miR‐125b mimics (*p* < 0.01). In short, these data strongly suggest that Linc‐smad7 directly sponges miR‐125b in HCC cells.

### 3.6 Linc‐smad7 promotes the proliferation, migration, invasion and EMT of HCC cells by directly targeting miR‐125b.

**Figure 5:**
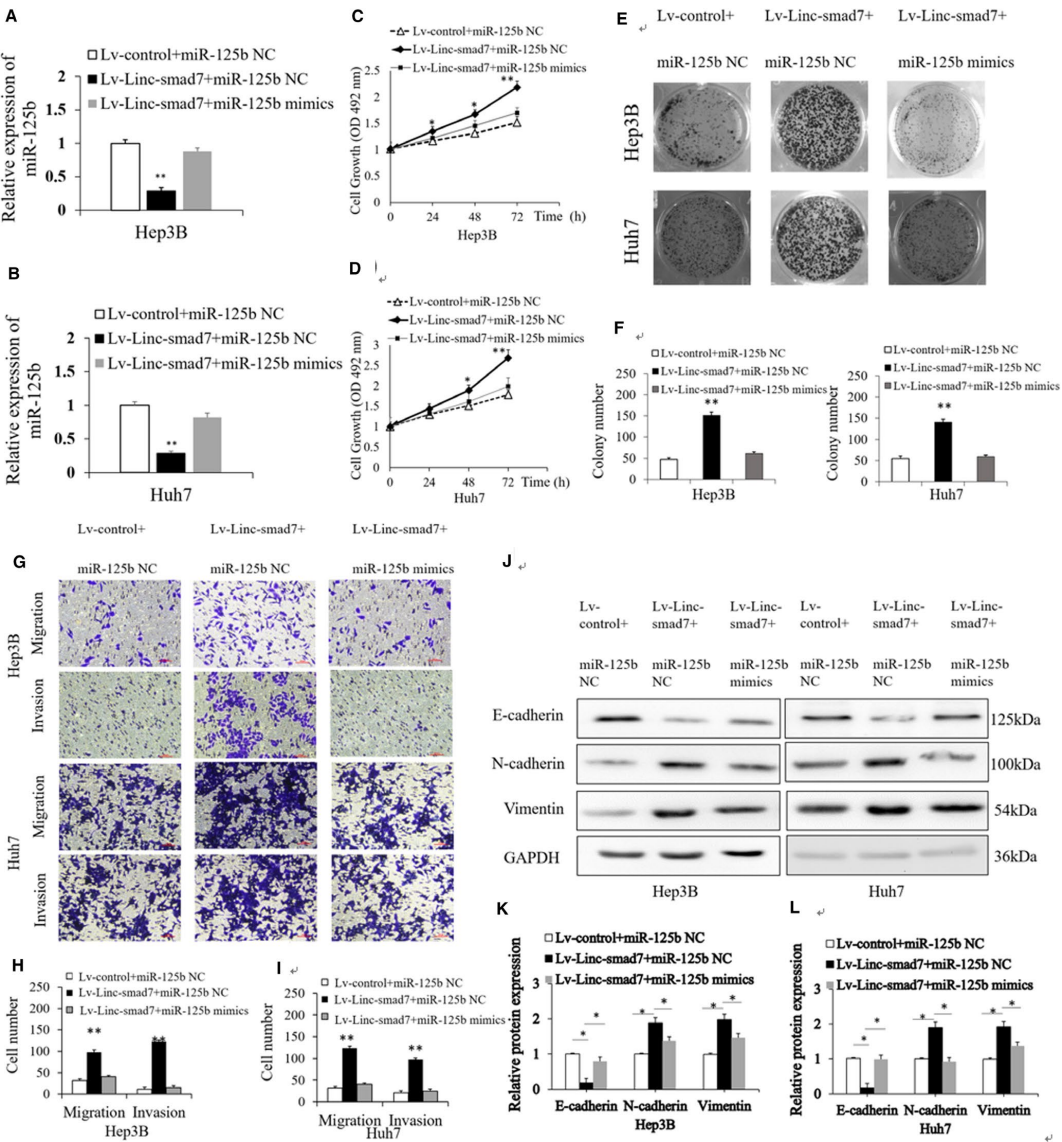
Upregulation of miR‑125b reverses the effects of Linc‐smad7 on the proliferation, migration, invasion and EMT of HCC cells. (A,B) miR‐125b mRNA expression was measured by RT‐qPCR assay (**p < 0.01). (C,D) MTT assay showed that overexpression of miR‐125b rescued the effect of Linc‐smad7 on HCC cell proliferation (*p < 0.05, **p < 0.01). (E,F) Overexpression of miR‐125b rescued the promoting effect of Linc‐smad7 on HCC cell colony formation (**p < 0.01). (G,H,I) Overexpression of miR‐125b rescued the promoting effect of Linc‐smad7 on HCC cell migration and invasion (**p < 0.01). (J,K,L) Overexpression of miR‐125b rescued the effect of Linc‐smad7 on HCC EMT (*p < 0.05).

To investigate whether Linc‐smad7 promotes the malignant behaviours of HCC cells by binding to miR‐125b, we assessed the combined effects of overexpression of Linc‐smad7 and miR‐125b on proliferation, invasion and migration of HCC cells. We transfected miR‐125b mimics into Hep3B and Huh7 cells overexpressing Linc‐smad7 and found the recovery of miR‐125b expression following co‐transfection with miR‐125b mimics (Figure 5A and B). MTT assay, colony formation assay and Transwell assays were performed, and the results showed that the promoting effects of Linc‐smad7 on the proliferation, migration and invasion of Hep3B and Huh7 cells were attenuated by co‐transfection of miR‐125b mimics (*p* < 0.05; Figure 5C‐I). The results of Western blot assay showed increased miR‐125b expression inhibited N‐cadherin and Vimentin expression, while E‐cadherin expression significantly upregulated in HCC cells overexpressing Linc‐smad7 (*p* < 0.05, Figure 5J‐L). These data suggested that Linc‐smad7 promoted HCC cells malignant biological behaviour by directly targeting miR‑125b.

### 3.7 Silencing SIRT6 attenuates the promoting effects of Linc‐smad7 on the proliferation, invasion, migration and EMT of HCC cells

**Figure 6:**
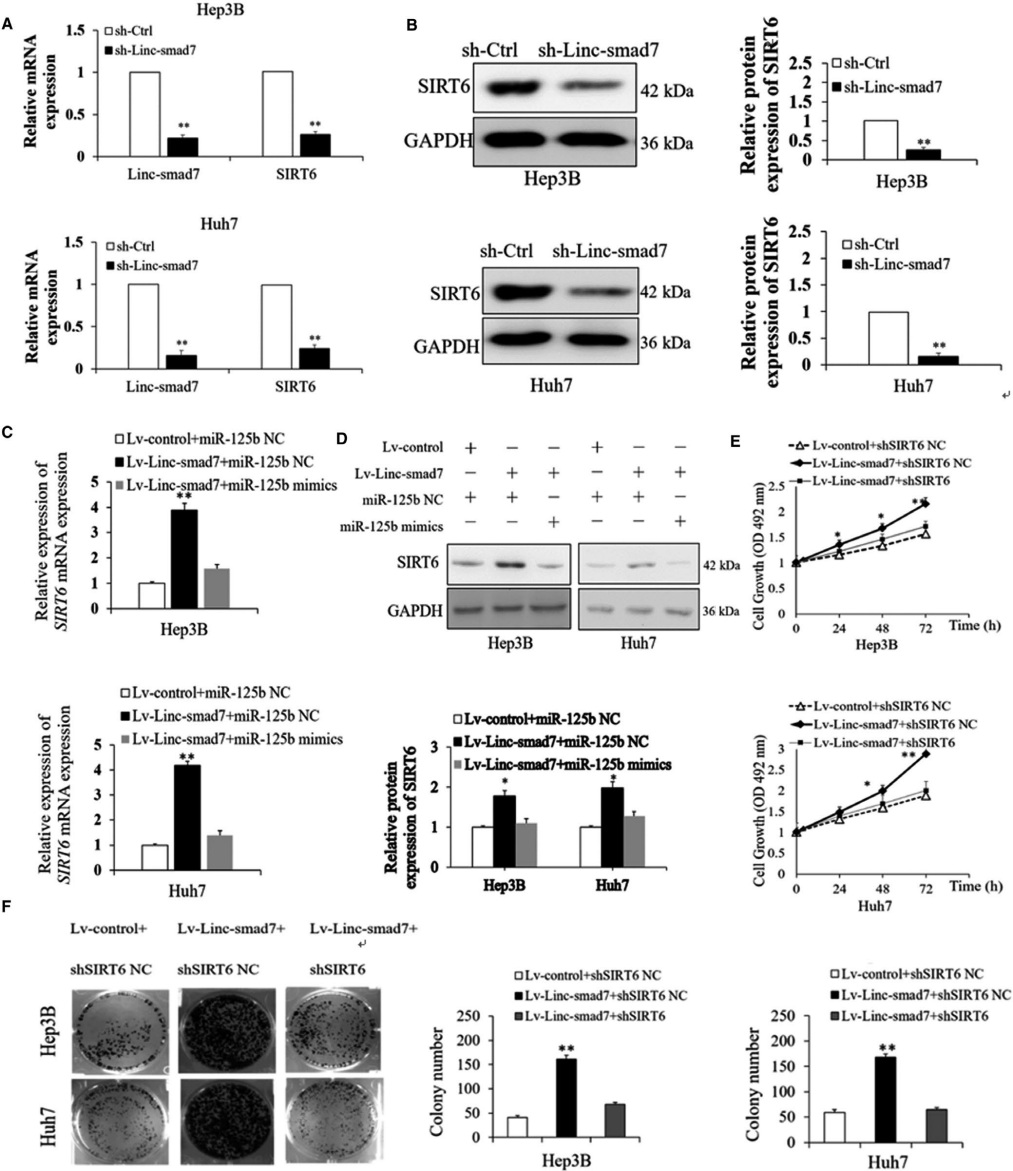
Silencing SIRT6 rescued the promoting effects of Linc‐smad7 on the proliferation of HCC cells. (A) Linc‐smad7 and SIRT6 mRNA expression was measured by RT‐qPCR assay (**p < 0.01). (B) Linc‐smad7 and SIRT6 protein expression was measured by western blotting assay (**p < 0.01). (C) SIRT6 mRNA expression was measured by RT‐qPCR assay (**p < 0.01). (D) SIRT6 protein expression was measured by western blotting assay (**p < 0.01). (E) MTT assay showed that knockdown of SIRT6 rescued the effect of Linc‐smad7 on HCC cells proliferation (*p < 0.05, **p < 0.01). (F) Knockdown of SIRT6 rescued the promoting effect of Linc‐smad7 on HCC cells colony formation (**p < 0.01).

**Figure 7:**
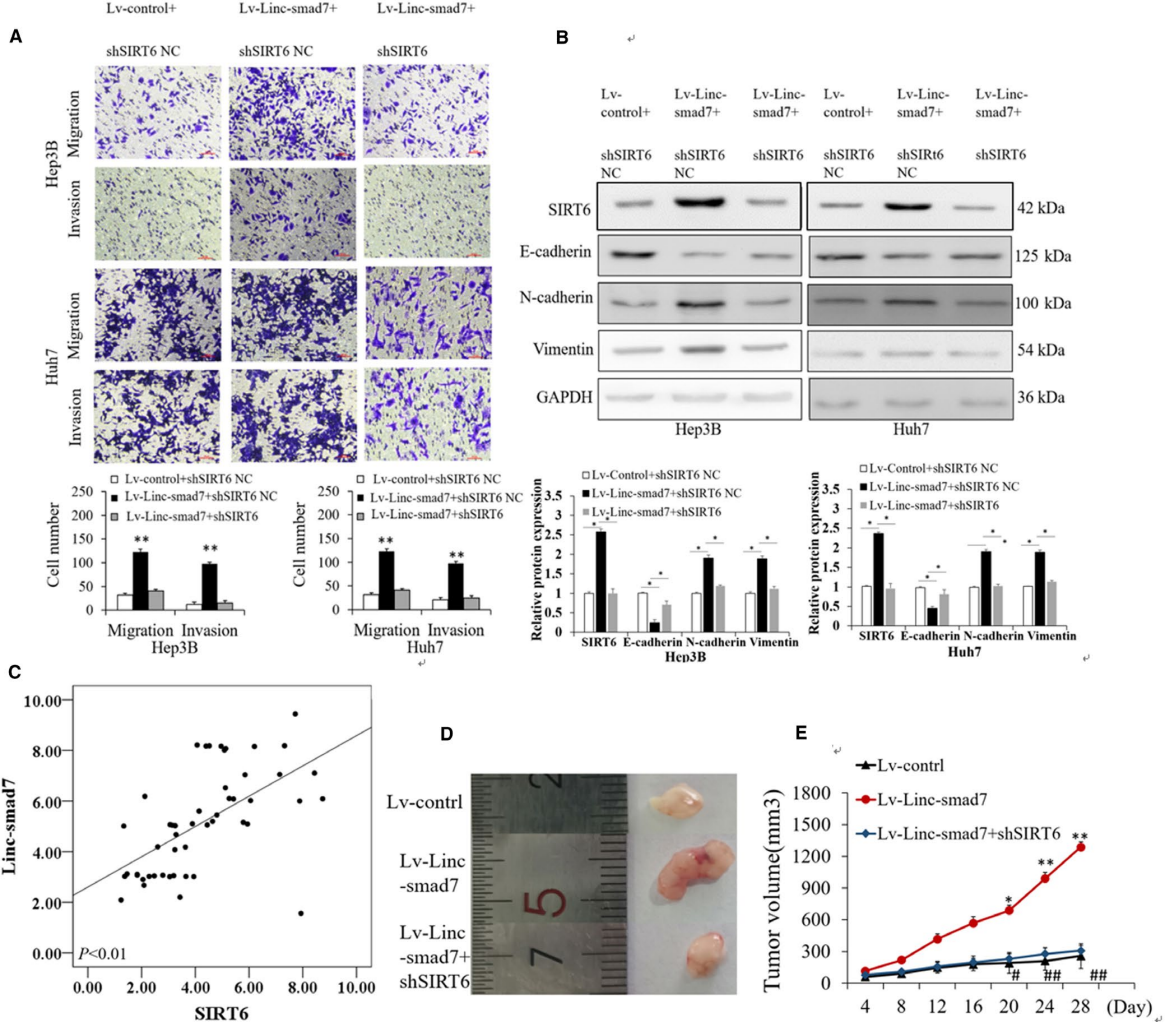
Silencing SIRT6 rescued the promoting effects of Linc‐smad7 on the migration, invasion and EMT of HCC. (A) Knockdown of SIRT6 rescued the promoting effect of Linc‐smad7 on HCC cell migration and invasion (**p < 0.01). (B) Knockdown of SIRT6 rescued the effect of Linc‐smad7 on HCC EMT (*p < 0.05). (C) The correlation of SIRT6 and Linc‐smad7 levels (*r* = 0.588; *p* < 0.01). (D,E) Increased Linc‐smad7 expression promoted HCC xenograft tumour growth (*p* < 0.05), while SIRT6 knockdown counteracted the effect of SIRT6 (*p* < 0.05).

A previous study suggested that SIRT6 may act as the downstream target of miR‑125b in HCC.^24^ Therefore, we hypothesized that the effects of Linc‐smad7 in HCC may be mediated via SIRT6. The results of RT‐qPCR and Western blot assay revealed that SIRT6 expression was decreased in Hep3B and Huh7 cells transfected with sh‐Linc‐smad7 vector compared with that in cells transfected with sh‐Ctrl vector (*p* < 0.01; Figure 6A and B). The upregulation of Linc‐smad7 enhanced the mRNA and protein expression levels of SIRT6, while the treatments of miR‐125b mimics partly reversed SIRT6 expression changes induced by overexpressed Linc‐smad7 (Figure 6C, D). These results suggested that Linc‐smad7 promoted SIRT6 expression upon miR‐125b. We subsequently knocked down SIRT6 expression via lentivirus transfection in HCC cells overexpressing Linc‐smad7. MTT assay and colony formation results showed that the proliferation capacity of Hep3B and Huh7 cells transfected with Lv‑Linc‐smad7 was restored following the knockdown of SIRT6 (Figure 6E and F **)**. In addition, Transwell assay results demonstrated that SIRT6 knockdown rescued the increased migration and invasion induced by Linc‐smad7 overexpression in Hep3B and Huh7 cells (Figure 7A). SIRT6 knockdown inhibited N‐cadherin and Vimentin expression but significantly enhanced E‐cadherin expression in HCC cells with Linc‐smad7 overexpression (*p* < 0.05, Figure 7B). Spearman correlation analysis using the RT‐qPCR results showed that Linc‐smad7 expression was positively associated with SIRT6 in HCC tissues (*r* = 0.588, *p* < 0.001, Figure 7C). At last, we established an HCC xenograft model using nude mice to verify our data in vivo, and found that Hep3B cells overexpressing Linc‐smad7 led to markedly larger tumours (Lv‐Linc‐smad7 group) than control vector cells (Lv‐control group) (*p* < 0.05, Figure 7D and E). Hep3B cells co‐transfected with Linc‐smad7 overexpression vector and sh‐SIRT6 vector generated smaller tumours (Lv‐Linc‐smad7+shSIRT6 group) than Hep3B cells only transfected with the Linc‐smad7 overexpression vector (*p* < 0.05, Figure 7D and E). In conclusion, these results suggest that Linc‐smad7 facilitates HCC cells proliferation, invasion and migration via miR‑125b /SIRT6 axis.

## 4 DISCUSSION

This study uncovered for the first time that Linc‐smad7 promoted the malignant biological behaviour of HCC by targeting the miR‑125b/SIRT6 axis. In the current study, the data revealed that Linc‐smad7 expression is upregulated in HCC tissues and cells.

Furthermore, increased Linc‐smad7 expression was demonstrated to be closely associated with poor clinicopathological features, shorter OS and DFS in HCC patients. In addition, Linc‐smad7 overexpression promoted HCC cells proliferation, migration, invasion and EMT. These data strongly demonstrate that Linc‐smad7 functions as a protumorigenic factor and therapeutic target in HCC. Numerous lncRNAs have reported to act as critical prognostic biomarkers and regulators in a variety of cancers.^25^, ^26^, ^27^ A recently published study identified Linc‐smad7, which is located adjacent to the mouse Smad7 gene, as a novel long non‐coding RNA via inhibiting the apoptosis of mouse breast cancer cells.^28^ Song et al. focused on the function of Linc‐smad7 in myoblast differentiation and confirmed the important role of Linc‐smad7 in promoting myoblast growth and muscle regeneration by enhancing the expression of smad7 and inhibiting miR‐125b.^24^ However, the expression profile and potential function of Linc‐smad7 in human cancers have not been studied before. Increased Linc‐smad7 expression and its positive association with poor clinicopathological features and prognosis in HCC patients were observed in the present study, suggesting that Linc‐smad7 may be promising biomarker and may act as a tumour promoter in HCC. To further study the role of Linc‐smad7 acted during HCC progression, we detected the malignant biological behaviour of HCC cells after Linc‐smad7 was overexpressed. Combined with the tests in clinical samples, the results in vitro demonstrated that Linc‐smad7 overexpression promoted the proliferation, migration, invasion and EMT of HCC cells. Our results further demonstrated that Linc‐smad7 serves as a protumourigenic factor in HCC.

A group of studies reveal that lncRNAs are able to function as ceRNAs through their miRNA response elements to interfere with miRNAs and their target mRNAs.^29^, ^30^, ^31^ Therefore, lncRNA‐miRNA‐mRNA interactional networks have been confirmed to be an important mechanism underlying the carcinogenesis and development of different types of cancer,^32^, ^33^, ^34^ including HCC.^35^ In the current study, miR‑125b was identified as a targeting miRNA that possesses binding sites for Linc‐smad7. Subsequently, our data demonstrated that Linc‐smad7 repressed miR‐125b expression in HCC cells, thus enhancing the expression of SIRT6, which is the miR‐125b target gene, ultimately promoting HCC cell proliferation, invasion, migration and EMT. Thus, the role of miR‑125b in the function of Linc‐smad7 in HCC was then investigated. In recent years, emerging studies have reported that miR‐125b plays a crucial role in HCC progression. Wang et al. reported that miR‐125b suppressed HCC cell proliferation and stem cell properties by targeting CD90, which is a marker of HCC stem cells.^36^ Similar findings in another study identified miR‐125b as a tumour suppressor of HCC that inhibits thioredoxin reductase 1 (TXNRD1).^37^ In addition, miR‐125b has been elucidated to reverse oxaliplatin resistance in HCC by inhibiting autophagy via regulating transmembrane protein 166 (TMEM 166).^38^ The results of the present study also demonstrated that restoring the expression of miR‑125b or knocking down SIRT6 expression reversed the promoting effects of Linc‐smad7 on the malignant phenotypes of HCC cells. Previous reports and the results of the present study support our hypothesis that Linc‐smad7 inhibited miR‐125b, a tumour suppressor in HCC, thereby exerting its tumour promoting function in HCC.

To further reveal the mechanism of Linc‐smad7 in HCC, we continued to explore its effect on target genes of miR‐125b. In the previously mentioned study, it revealed that decreased miR‑125b was closely correlated with a poor prognosis of HCC patients, and miR‐125b can function as a cancer suppressor by targeting SIRT6.^24^ SIRT6, which belongs to the sirtuin family, is an NAD+‐dependent deacetylase that plays a pivotal role in human cancers by targeting a variety of proteins.^39^, ^40^

Numerous previous studies have confirmed that SIRT6 can function as an oncogene in skin squamous cell carcinoma, multiple myeloma and acute myeloid leukaemia.^41^, ^42^, ^43^ Most recently, one of our studies demonstrated that SIRT6 promotes HCC metastasis by enhancing E‐cadherin autophagic degradation via Beclin‐1 deacetylation.^44^ Several other independent studies also confirmed the important protumorigenic factor role of SIRT6 in HCC carcinogenesis. Zhang et al. uncovered that SIRT6 enhances the proliferation capacity of HCC cells via the ERK1/2 signalling pathway.^45^ Ran et al. reported that SIRT6 inhibited HCC cell apoptosis by suppressing the binding of transcription factors to the promoter of BCL2‐associated X protein (Bax).^46^ Conclusively, these data support the conclusion that linc‐smad7 promotes HCC tumourigenesis mainly by targeting the miR‑125b/SIRT6 axis, although other possible mechanisms were not explored.

To summarize, this study identified the lncRNA linc‐smad7 as a novel potential oncogene in HCC. Our observations revealed that linc‐smad7 overexpression dramatically promoted HCC cell proliferation, migration and invasion. At the molecular level, the current study demonstrated that Linc‐smad7 directly inhibited miR‑125b, and subsequently enhanced SIRT6 expression. Furthermore, we demonstrated that miR‑125b overexpression or SIRT6 knockdown rescued the protumourigenic effects of Linc‐smad7 overexpression on HCC cells. The present study has provided novel insight into the regulatory mechanisms involving Linc‐smad7 and the miR‑125b/SIRT6 axis in HCC tumourigenesis and progression, may provide a novel target for the clinical treatment of HCC in the future.

## CONFLICT OF INTEREST

All authors have no conflict of interest.

## Supporting information

Figure S1.

Figure S2

Figure S3.

## Data Availability

The datasets used during the present study are available from the corresponding author upon reasonable request.

## References

[1] Llovet JM , Zucman‐Rossi J , Pikarsky E , Sangro B , Schwartz M , Sherman M . Hepatocellular carcinoma. Nat Rev Dis Primers. 2016;2:16018. 27158749 10.1038/nrdp.2016.18

[2] Galun D , Srdic‐Rajic T , Bogdanovic A . Targeted therapy and personalized medicine in hepatocellular carcinoma: drug resistance, mechanisms, and treatment strategies. J Hepatocell Carcinoma. 2017;4:93‐103. 28744453 10.2147/JHC.S106529 PMC5513853

[3] Forner A , Liovet JM , Bruix J . Hepatocellular carcinoma. Lancet. 2013;79:1245‐1255. 10.1016/S0140-6736(11)61347-0 PMC13537732 22353262

[4] Kalluri R , Weinberg RA . The basics of epithelial‐mesenchymal transition. J Clin Invest. 2009;119:1420‐1428. 19487818 10.1172/JCI39104 PMC2689101

[5] Niinaka Y , Harada K , Fujimuro M , et al. Silencing of autocrine motility factor induces mesenchymal‐to‐epithelial transition and suppression of osteosarcoma pulmonary metastasis. Cancer Res. 2010;70:9483‐9493. 20978190 10.1158/0008-5472.CAN-09-3880 PMC2982894

[6] Lv YF , Dai H , Yan GN , Meng G , Zhang X , Guo QN . Downregulation of tumor suppressing STF cDNA 3 promotes epithelial‐mesenchymal transition and tumor metastasis of osteosarcoma by the Wnt/GSK‐3beta/beta‐catenin/snail signaling pathway. Cancer Lett. 2016;373:164‐173. 26845447 10.1016/j.canlet.2016.01.046

[7] De Craene B , Berx G . Regulatory networks defining EMT during cancer initiation and progression. Nat Rev Cancer. 2013;13:97‐110. 23344542 10.1038/nrc3447

[8] Lamouille S , Xu J , Derynck R . Molecular mechanisms of epithelial‐mesenchymal transition. Nature Reviews Mol Cell Biol. 2014;15:179‐196. 10.1038/nrm3758 PMC4240281 24556840

[9] Fatica A , Bozzoni I . Long non‐coding RNAs: new players in cell differentiation and development. Nat Rev Genet. 2014;15:7‐21. 24296535 10.1038/nrg3606

[10] Mercer TR , Dinger ME , Mattick JS . Long non‐coding RNAs: Insights into functions. Nat Rev Genet. 2009;10:155‐159. 19188922 10.1038/nrg2521

[11] Li J , Xuan Z , Liu C . Long non‐coding RNAs and complex human diseases. Int J Mol Sci. 2013;14:18790‐18808. 24036441 10.3390/ijms140918790 PMC3794807

[12] Gao Y , Zhang Z , Li K , et al. Linc‐DYNC2H1‐4 promotes EMT and CSC phenotypes by acting as a spongeof miR‐145 in pancreatic cancer cells. Cell Death Dis. 2017;13:e2924. 10.1038/cddis.2017.311 PMC5550858 28703793

[13] Chen W , Wang H , Liu Y , et al. Linc‐RoR promotes proliferation, migration, and invasion via the Hippo/YAP pathway in pancreatic cancer cells. J Cell Biochem. 2019;26. 10.1002/jcb.29308 31452251

[14] Guo L , Sun C , Xu S , et al. Knockdown of long non‐coding RNA linc‐ITGB1 inhibits cancer stemness and epithelial‐mesenchymal transition by reducing the expression of Snail in non‐small cell lung cancer. Thorac Cancer. 2019;10:128‐136. 30485693 10.1111/1759-7714.12911 PMC6360263

[15] Yu WW , Wang K , Liao GJ . Knockdown of long noncoding RNA linc‐ITGB1 suppresses migration, invasion of hepatocellular carcinoma via regulating ZEB1. Eur Rev Med Pharmacol Sci. 2017;21:5089‐5095. 29228420 10.26355/eurrev_201711_13823

[16] Yuan Z , Xiu C , Liu D , et al. Long noncoding RNA LINC‐PINT regulates laryngeal carcinoma cell stemness and chemoresistance through miR‐425‐5p/PTCH1/SHH axis. J Cell Physiol. 2019;234:23111‐23122. 31131448 10.1002/jcp.28874

[17] Han LL , Nan HC , Tao T , et al. Expression and significance of the novel tumor‐suppressor gene SMG‐1 in hepatocellular carcinoma. Oncol Rep. 2014;6:2569‐2578. 10.3892/or.2014.3125 24700316

[18] Sun L , Lian JX , Meng S . MiR‐125a‐5p promotes osteoclastogenesis by targeting TNFRSF1B. Cell Mol Biol Lett. 2019;28:23. 10.1186/s11658-019-0146-0 PMC6437974 30976285

[19] Chen YF , Wei YY , Yang CC , et al. miR‐125b suppresses oral oncogenicity by targeting the anti‐oxidative gene PRXL2A. Redox Biol. 2019;22:101140. 30785086 10.1016/j.redox.2019.101140 PMC6383183

[20] Zhou HC , Fang JH , Shang LR , et al. MicroRNAs miR‐125b and miR‐100 suppress metastasis of hepatocellular carcinoma by disrupting the formation of vessels that encapsulate tumour clusters. J Pathol. 2016;4:450‐460. 10.1002/path.4804 27577856

[21] Zhou JN , Zeng Q , Wang HY , et al. MicroRNA‐125b attenuates epithelial‐mesenchymal transitions and targets stem‐like liver cancercells through small mothers against decapentaplegic 2 and 4. Hepatology. 2015;3:801‐815. 10.1002/hep.27887 25953743

[22] Tsang FH , Au SL , Wei L , et al. Long non‐coding RNA HOTTIP is frequently up‐regulated in hepatocellular carcinoma and is targeted by tumour suppressive miR‐125b. Liver Int. 2015;5:1597‐1606. 10.1111/liv.12746 25424744

[23] Song C , Wang J , Ma Y , et al. Linc‐smad7 promotes myoblast differentiation and muscle regeneration via sponging miR‐125b. Epigenetics. 2018;6:591‐604. 10.1080/15592294.2018.1481705 PMC6140903 29912619

[24] Song S , Yang Y , Liu M , et al. MiR‐125b attenuates human hepatocellular carcinoma malignancy through targeting SIRT6. Am J Cancer Res. 2018;6:993‐1007. PMC6048406 30034937

[25] Gao H , Wang T , Zhang P , et al. Linc‐ROR regulates apoptosis in esophageal squamous cell carcinoma via modulation of p53 ubiquitination by targeting miR‐204‐5p/MDM2. J Cell Physiol. 2019;20. 10.1002/jcp.29139 31541467

[26] Zhang L , Chen J , Wang LI , et al. Linc‐PINT acted as a tumor suppressor by sponging miR‐543 and miR‐576‐5p in esophageal cancer. J Cell Biochem. 2019;28. 10.1002/jcb.28699 31464068

[27] He X , Yu J , Xiong L , et al. Exosomes derived from liver cancer cells reprogram biological behaviors of LO2 cells by transferring Linc‐ROR. Gene. 2019;30:144044. 10.1016/j.gene.2019.144044 31400406

[28] Arase M , Horiguchi K , Ehata S , et al. Transforming growth factor‐β‐induced lncRNA‐Smad7 inhibits apoptosis of mouse breast cancer JygMC (A) cells. Cancer Sci. 8(2014), pp. 974–982. 10.1111/cas.12454 PMC4317863 24863656

[29] Ye JR , Liu L , Zheng F . Long Noncoding RNA Bladder Cancer associated transcript 1 promotes the proliferation, migration, and invasion of nonsmall cell lung cancer through sponging miR‐144. DNA Cell Biol. 2017;10:845‐852. 10.1089/dna.2017.3854 28885863

[30] Liu W . LncRNA LINC‐PINT Inhibits Cancer Cell Proliferation, Invasion, and Migration in Osteosarcoma by Downregulating miRNA‐21. Cancer Biother Radiopharm. 2019;4:258‐263. 10.1089/cbr.2018.2684 31070482

[31] Lu SR , Li Q , Lu JL , Liu C , Xu X , Li JZ . Long non‐coding RNA LINC01503 promotes colorectal cancer cell proliferation and invasion by regulating miR‐4492/FOXK1 signaling. Exp Ther Med. 2018;6:4879‐4885. 10.3892/etm.2018.6775 PMC6257603 30542444

[32] Zhao J , Xu J , Wang W , et al. Long non‐coding RNA LINC‐01572:28 inhibits granulosa cell growth via a decrease in p27 (Kip1) degradation in patients with polycystic ovary syndrome. EBioMedicine. 2018;36:526‐538. 30293818 10.1016/j.ebiom.2018.09.043 PMC6197751

[33] Liu Q , Zhang W , Wu Z , et al. Construction of a circular RNA‐microRNA‐messengerRNA regulatory network in stomach adenocarcinoma. J Cell Biochem. 2019;4. 10.1002/jcb.29368 31486138

[34] Lyu L , Xiang W , Zhu JY , Huang T , Yuan JD , Zhang CH . Integrative analysis of the lncRNA‐associated ceRNA network reveals lncRNAs as potential prognostic biomarkers in human muscle‐invasive bladder cancer. Cancer Manag Res. 2019;11:6061‐6077. 31308745 10.2147/CMAR.S207336 PMC6614857

[35] Kouhsar M , Azimzadeh Jamalkandi S , Moeini A , Masoudi‐Nejad A . Detection of novel biomarkers for early detection of Non‐Muscle‐Invasive Bladder Cancer using Competing Endogenous RNA network analysis. Sci Rep. 2019;9:8434. 31182759 10.1038/s41598-019-44944-3 PMC6557814

[36] Wang Y , Wang B , Xiao S , Li Y , Chen Q . miR‐125a/b inhibits tumor‐associated macrophages mediated in cancer stem cells of hepatocellular carcinoma by targeting CD90. J Cell Biochem. 2019;3:3046‐3055. 10.1002/jcb.27436 30536969

[37] Hua S , Quan Y , Zhan M , Liao H , Li Y , Lu L . miR‐125b‐5p inhibits cell proliferation, migration, and invasion in hepatocellular carcinoma via targeting TXNRD1. Cancer Cell Int. 2019;19:203. 31384178 10.1186/s12935-019-0919-6 PMC6668076

[38] Ren WW , Li DD , Chen X , et al. MicroRNA‐125b reverses oxaliplatin resistance in hepatocellular carcinoma by negatively regulating EVA1A mediated autophagy. Cell Death Dis. 2018;9:547. 29749374 10.1038/s41419-018-0592-z PMC5945723

[39] Huang Z , Zhao J , Deng W , et al. In dentification of a cellularly active SIRT6 allosteric activator. Nat Chem Biol. 2018;14:1118‐1126. 30374165 10.1038/s41589-018-0150-0

[40] Cea M , Cagnetta A , Adamia S . Evidence for a role of the histone deacetylase SIRT6 in DNA damage response of multiple myeloma cells. Blood. 2016;127:1138‐1150. 26675349 10.1182/blood-2015-06-649970 PMC4778164

[41] Ming M , Han W , Zhao B , et al. SIRT6 promotes COX‐2 expression and acts as an oncogene in skin cancer. Cancer Res. 2014;74:5925‐5933. 25320180 10.1158/0008-5472.CAN-14-1308 PMC4203414

[42] Lefort K , Brooks Y , Ostano P , et al. A miR‐34a‐SIRT6 axis in the squamous cell differentiation network. EMBO J. 2013;32:2248‐2263. 23860128 10.1038/emboj.2013.156 PMC3746195

[43] Cagnetta A , Soncini D , Orecchioni S , et al. Depletion of SIRT6 enzymatic activity increases acute myeloid leukemia cells vulnerability to DNA‐damaging agents. Haematologica. 2018;103:80‐90. 29025907 10.3324/haematol.2017.176248 PMC5777193

[44] Han LL , Jia L , Wu F , Huang C . Sirtuin6 (SIRT6) Promotes the EMT of Hepatocellular Carcinoma by Stimulating Autophagic Degradation of E‐Cadherin. Mol Cancer Res. 2019;17:2267‐2280. 31551254 10.1158/1541-7786.MCR-19-0321

[45] Zhang C , Yu Y , Huang Q , Tang K . SIRT6 regulates the proliferation and apoptosis of hepatocellular carcinoma via the ERK1/2 signaling pathway. Mol Med Rep. 2019;20:1575‐1582. 31257493 10.3892/mmr.2019.10398 PMC6625461

[46] Ran LK , Chen Y , Zhang ZZ , et al. SIRT6 Overexpression Potentiates Apoptosis Evasion in Hepatocellular Carcinoma via BCL2‐Associated X Protein‐Dependent Apoptotic Pathway. Clin Cancer Res. 2016;13:3372‐3382. 10.1158/1078-0432.CCR-15-1638 26861461

