# Supplementary figures and images for: Long intergenic noncoding RNA smad7 (Linc‐smad7) promotes the epithelial‐mesenchymal transition of HCC by targeting the miR‑125b/SIRT6 axis
Source: Cancer Med. 2020 Oct 10;9(23):9123–37. doi: 10.1002/cam4.3515 (PMC7724296; doi:10.1002/cam4.3515)

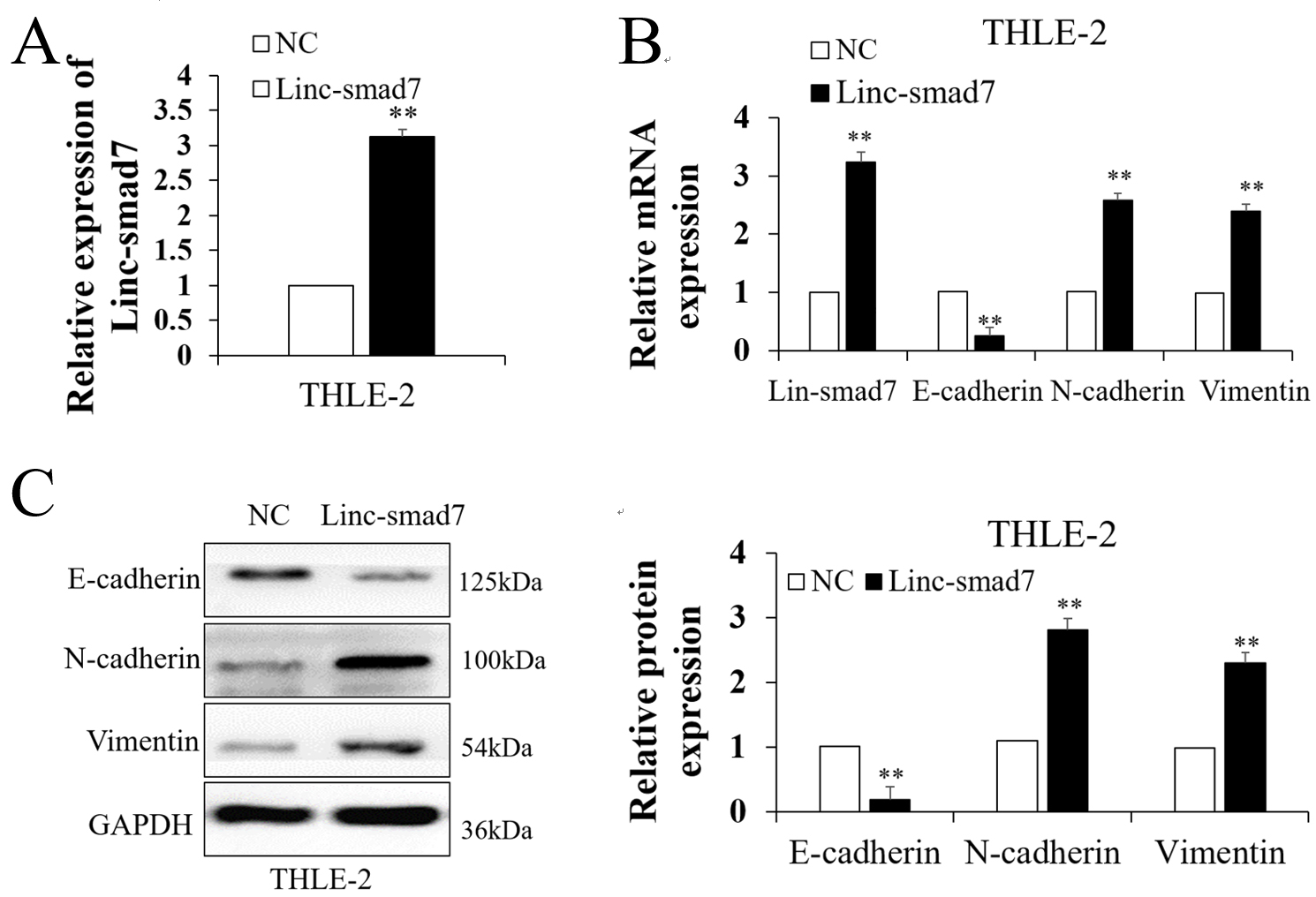

Supplement: Supplementary file 1 — Figure S1. [file CAM4-9-9123-s001.jpg]

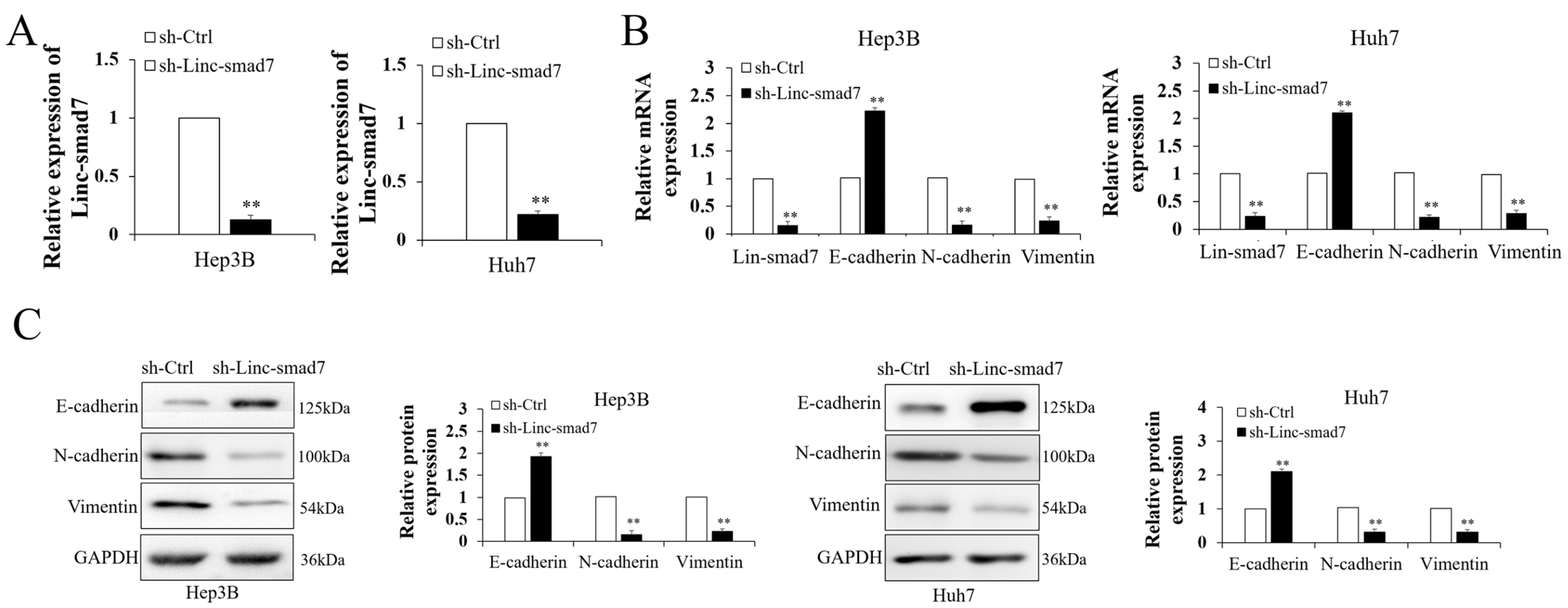

Supplement: Supplementary file 2 — Figure S2 [file CAM4-9-9123-s002.jpg]

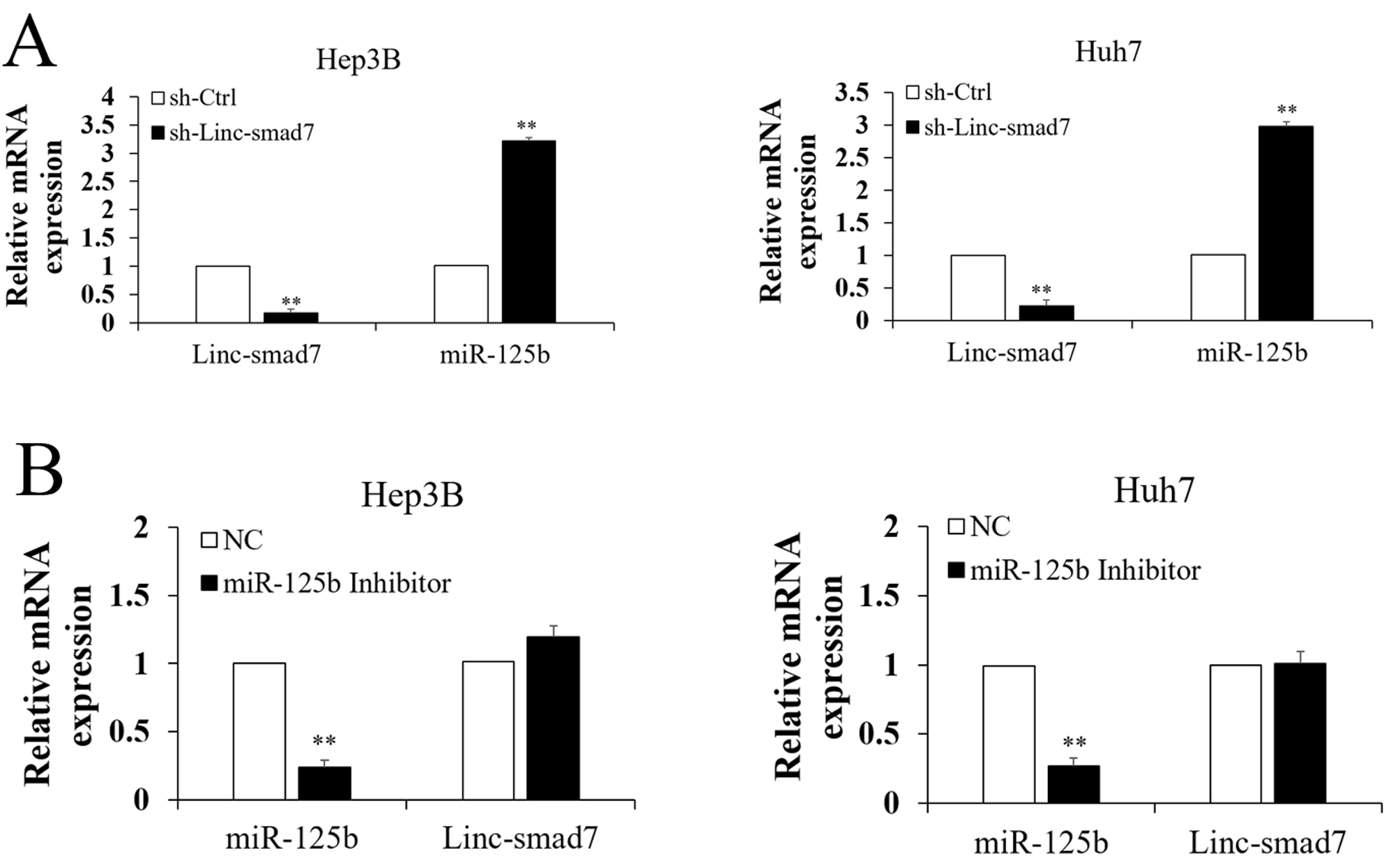

Supplement: Supplementary file 3 — Figure S3. [file CAM4-9-9123-s003.jpg]
